# Scotch Physicians and Surgeons

**Published:** 1888-04-07

**Authors:** C. G. Furley


### Scotch Physicians and Surgeons.

By C. G. Furley.

*(Continued from p. 427, vol. iii.)*

### SIR ROBERT CHRISTISON.

When the Volunteer movement began in 1859 Christison interested himself greatly in it, and soon took a prominent part. It was he who encouraged the students of the Univerpity to form a corps, and himself commanded it till he was seventy-seven years of age. In this semi-military position he proved himself a strict disciplinarian, but he was proud to boast of the superiority of "his lads" to other Volunteer regiments, and even to compare them not unfavourably with some of the "regulars." When he retired from his command, in which he was succeeded by Professor (now Sir William) Turner, he was presented with a sword of honour from the present and past members of his corps, a compliment which touched him deeply.

Among other honours that came to him in his later years was the baronetcy offered him by Mr. Gladstone in 1871. As Christison was a staunch Conservative in all things, social and political, the honour, coming through such hands, was a valued testimony to his professional qualities, and could be proffered from no other consideration. In the following year the University of Edinburgh gave him the degree of LL.D., making an exception to the rule in force there of not granting an honorary degree to its own graduates.

This, however, was the only special honour the University he loved bestowed on him; the highest post connected with it escaped his grasp. During the long illness of Sir David Brewster, Christison fulfilled the duties connected with the principalship, and it was almost taken for granted that he would inherit the post when Brewster died; even Simpson waiving the claims his exceptional distinction gave him in favour of Christison's superior fitness. "Dr. Christison is the man," he said, when urged to seek the post. When it came to the point, however, other candidates appeared; Simpson also entered the contest; and Christison, who would have valued the courtesy implied in an unopposed election, but had no liking for going about asking for votes, practically withdrew. As has been said, Sir Alexander Grant was chosen, possibly—let us hope so—because the electors felt it would be invidious to choose either Simpson or Christison in preference to the other.

Another post, which he would probably have valued even more, was denied him. In 1880 he was asked to become a candidate for the position of Lord Rector of the University; the other candidate being Lord Rosebery. A few years before, Christison's election would have been assured; but the Rector is chosen by the students, a race whose generations fleet quickly by; and those whom he had taught had now gone to take their part in life's work, and were replaced by others who knew not Christison. Again, had they been all natives of Edinburgh, even of Scotland, where his name was known and honoured, he might have been chosen; but the Edinburgh students come from every quarter of the globe.

Had academic considerations been predominant, there is no doubt that again "Sir Robert Christison was the man"; but these were at a discount just then, while political feeling ran high; the election became a contest, not between two men, but between two parties, and Lord Rosebery was elected.

It was a little hard; more than a little unwise. Lord Rosebery could not give the close and affectionate attention to University interests which Christison would have rendered. These always stood first with him, though we may hold that he did not always seek them wisely. Such at least will be the opinion of many people in regard to his opposition to the admission of lady students to the advantages of medical education within its walls. Although he did not see that there was any special necessity for women doctors in this country, he approved of their studying with a view to practice in India; and thought that obstetrics ought to be generally left to them everywhere. "If ladies would espouse this branch of medical practice their success would be a great blessing to the public," he says; but if admitting them to his beloved University was to lessen the influx of male students, on whom its prosperity depended, he would none of them. Beyond this, moreover, he held a chivalrous but somewhat narrow view of woman's place in the world—a view not unpleasing to women of wealth and leisure, and clung to with determined helplessness by idle and incompetent women of all classes; but one which the most active-minded of our day are very determined to prove erroneous. With Sir Robert Christison, however, it sprang from a hidden tenderness which those who knew him only in his more austere official aspect did not suspect him of, but which shows itself in his autobiography in one or two allusions necessary in telling of his professional career, to "what should otherwise," he considers, "be inviolable." Very slight and reverent are these allusions, and we may supplement them by an anecdote narrated by Dr. John Brown, which shows the real tender soul within the man:

"His wife (who died in 1849), a woman of great beauty, *and better*, was in her last long illness. She was going to the country for a month, and her husband heard her give orders that a piece of worsted work which she had finished should be *grounded* (ladies will understand what this word means), and made up as an ottoman before her return. A few days before she came back he asked if the work was completed; it had been totally forgotten. He said nothing; but getting possession of the piece he sat up for two or three nights and grounded it with his own hands; had it made up, and when his wife came home set her down on it as she had wished."

Very foolish and sentimental this, is it not, O practical men of the world? You would not grudge your wives anything necessary for their health or comfort, nay, any luxury they chose to ask for; but this throwing away of necessary rest and wasting time in trivial unfamiliar women's needlework is a different thing, unworthy of your manly common-sense. Perhaps so; and yet from the dying wife's point of view the task was neither foolish nor unmanly; one of the deeds that have no market price, because so far beyond our worldly valuing.

For several years after his active career ceased Sir Robert Christison's tall figure, carrying erectly the burden of eighty years, was a familiar one in Edinburgh. The light, quick step that had carried him up so many hills since that first expedition in his student days that had imbued him with a passion for mountaineering, was but little feebler than in his prime, when it had been his habit to hasten the departure of his occasional attacks of fever by violent physical exercise. He climbed his last hill on the 5th September, 1881. It was a height of 1,250 feet, and rough ground, but he felt no extreme fatigue. During the autumn, however, a cold developed seriously; the strength that could go on well enough when there was no special strain upon it could not resist a shock. After a few months of weakness, he died on January 27th, 1882.

Sir Robert Christison's character was not one that attracted everyone. He was not, on the whole, a broad-minded man; he had strong prejudices, and he was very proud; but he was honourable in the utmost degree, and he was unselfishly loyal to the objects he cared for and the work he undertook. His private practice might have been large and remunerative long before it was so, if he could have persuaded himself to—not neglect—but give a less engrossing attention to his University duties. But that would have been impossible to him. A duty undertaken must be fulfilled to the best of his ability. He belonged in his ideals and his opinions to a former generation, less cosmopolitan than ours but not less noble; often, perhaps, nobler in its austerity than our all-round sympathies—with our own defects as well as our neighbours'—enable us to be; and he may be best defined by the title Dr. John Brown gave him—*Ultimus Romanorum.*

